# The role of adipose tissue and adipokines in the manifestation of type 2 diabetes in the long-term period following myocardial infarction

**DOI:** 10.1186/s13098-016-0136-6

**Published:** 2016-03-17

**Authors:** Olga Barbarash, Olga Gruzdeva, Evgenya Uchasova, Yulia Dyleva, Ekaterina Belik, Olga Akbasheva, Victoria Karetnikova, Alexander Kokov

**Affiliations:** Federal State Budgetary Institution “Research Institute for Complex Issues of Cardiovascular Disease”, Kemerovo, Russia; Laboratory of Research Homeostasis, Federal State Budgetary Institution “Research Institute for Complex Issues of Cardiovascular Disease”, Kemerovo, Russia; Department of Biochemistry and Molecular Biology, State Budget Educational Institution of Higher Professional Education “Siberian State Medical University” of the Russian Federation Ministry of Health, Tomsk, Russia; Laboratory of Blood Circulation Pathology, Federal State Budgetary Institution “Research Institute for Complex Issues of Cardiovascular Disease”, Kemerovo, Russia; Laboratory and X-ray tomographic diagnosis, Federal State Budgetary Institution “Research Institute for Complex Issues of Cardiovascular Disease”, Kemerovo, Russia

**Keywords:** Carbohydrate metabolism disorders, Myocardial infarction, Inflammation percutaneous coronary intervention, Adipose tissue, Diabetes, Insulin resistance, Adipokines, Myocardial infarction

## Abstract

**Background:**

This study aimed to evaluate the markers of insulin resistance and adipokine status in patients with visceral obesity during hospitalization following myocardial infarction (MI) and assess the disturbances of carbohydrate metabolism present 1 year after MI onset.

**Methods:**

94 male patients with MI were recruited. The exclusion criteria were as follows: age less than 50 or greater than 80 years, the presence of type 2 diabetes mellitus (T2DM), and a prior history of pronounced renal failure.Obesity types were defined according to body mass index (BMI), waist circumference (WC) and visceral adipose tissue (VAT) area. Glucose, insulin, adiponectin, leptin, and insulin resistance (IR) index were measured on days 1 and 12 after the onset of MI. New-onset type 2 diabetes was assessed 1 year after MI onset.

**Results:**

According to computed tomography assessments of all study patients, 69 % of patients with MI suffered from visceral obesity. The VAT area was more closely associated with the risk of developing type 2 diabetes compared with the obesity parameters, BMI and WC. Patients with a VAT area greater than 130 cm^2^ had a 3.6-fold higher risk of developing type 2 diabetes. The presence of IR and hyperleptinemia increased the risk of developing diabetes in the post-MI period 3.5 and 3.7 times, respectively, in patients with visceral obesity compared with patients without visceral obesity.

**Conclusion:**

Visceral obesity is associated with IR, a 5.7-fold increase in leptin levels and a high risk of developing type 2 diabetes 1 year after MI onset.

## Background

Currently, obesity is recognized as one of the primary elements in the pathogenesis of cardiovascular disease (CVD) and type 2 diabetes mellitus (T2DM) [1]. Visceral fat is reported to be far more dangerous than any other form of obesity [2]. Visceral adipose tissue (VAT) is known to be an independent risk factor of myocardial infarction (MI) in elderly patients (55/60–74 years) or patients at an advanced age (75–90) [3]. Recently, a relationship between excessive accumulation of adipose tissue in the visceral depot and progression of heart failure in patients after MI was reported [4].

Body mass index (BMI) is a traditional measure of obesity. Although BMI values may be within the normal range, patients may still have excessive abdominal adipose tissue, and thus, are at high risk of MI. Waist circumference (WC) is considered to be an indicator of abdominal obesity, but it has not been found to be a sufficiently accurate indicator of the risk of developing CVD and T2DM [5]. Computed tomography (CT), however, can be used in clinical practice to measure visceral fat [6].

VAT is not only a storage organ for excess energy; rather, it is an active endocrine organ responsible for the synthesis and secretion of hormone-like substances known as adipokines [7]. Adipokines have diverse roles in regulating different metabolic processes in the body, ranging from food intake to nutrient recycling at a molecular level [4]. Reportedly, an excess of VAT is associated with increased release of adipokines, contributing to the development of insulin resistance (IR) [3]. It has been hypothesized that during the occurrence of MI, catecholamine stress can worsen IR and may lead to the development of diabetes. The aim of this study was to investigate the markers of IR and adipokine status in patients with visceral obesity during hospitalization following MI and to assess the disturbances of carbohydrate metabolism 1 year after MI.

## Methods

### Study population

Ninety four male patients who were admitted to our hospital for MI between January 2008 and December 2010 were recruited, and the diagnosis of MI was verified using the All-Russian Scientific Society of Cardiology (2007) and European Society of Cardiology (ESC), the American College of Cardiology Foundation (ACCF), the American Heart Association (AHA), and the World Heart Federation (WHF) [8] diagnostic criteria for the diagnosis of MI: i.e., the presence of typical pain lasting longer than 15 min, electrocardiographic (ECG) changes (ST segment elevation in at least two consecutive leads) and laboratory findings (elevated creatine phosphokinase [CK], creatine phosphokinase-MB [CK-MB], and troponin T levels). The exclusion criteria were as follows: age less than 50 or greater than 80 years, the presence of T2DM, and a prior history of pronounced renal failure (glomerular filtration rate <30 mL/min). We recruited 33 participants without CVD or T2DM as controls. These participants were comparable to the enrolled patients in terms of age and sex.

The examination scheme and clinical assessment of patients included the collection of patient complaints and past medical history; physical examination; ECG recording; measurement of body weight (kg), height (cm), WC and hip circumference (HC) (cm); and calculation of BMI (kg/cm^2^) and waist-to-hip ratio. All the patients underwent multi-slice computed tomography on a computed tomography (Lightspeed VCT 64, General Electric, Fairfield, CT, USA) for the measurement of abdominal adipose tissue. The CT scans were performed at the L4 and L5 level, with the patients lying flat on their backs, with their hands raised, and holding their breath. A reconstruction slice thickness of 1 mm was used to reduce the effect of summation of adjacent tissue densities. VAT and subcutaneous adipose tissue (SAT) areas were measured, as well as the ratio of VAT to SAT. Two diagnostic criteria were used to confirm visceral obesity: a VAT area above 130 cm^2^ and a ratio of VAT to SAT above 0.4 [8]. Patients were categorized into two groups according to the CT findings: Group 1 (n = 64) included patients with visceral obesity (VAT area ≥130 cm^2^ and VAT/SAT ratio ≥0.4), and Group 2 (n = 30) included patients without visceral obesity (VAT area <130 cm^2^ and VAT/SAT ratio <0.4).

All the patients underwent primary percutaneous coronary intervention of the infarct-related artery as reperfusion therapy. During the hospitalization period (mean duration of 12 days), all the patients received β-blockers, angiotensin converting enzyme inhibitors, calcium channel blockers, diuretics, nitrates, aspirin, heparin, clopidogrel and statins.

The homeostasis model assessment method for evaluating IR (HOMA-IR) was used to assess IR. The HOMA-IR was calculated using the following formula: HOMA-IR = [fasting insulin (mkME/ml) × fasting glucose (mmol/l)]/22.5. A value of HOMA-IR above 2.77 implied the presence of abnormally high IR.

### Glucose tolerance test

All the patients (on day 12) underwent a 75-g oral glucose tolerance test (OGTT) according to specialized medical care standards to verify the diagnosis of T2DM. Blood glucose was measured after an overnight fast of 8–14 h and 2 h after a standard glucose load of 75 g (dissolved in 300 ml of water). Three days before the test, the patients were on an arbitrary (unbounded) diet. They maintained adequate physical activity according to the individual patient’s ability. The last evening meal contained 30–50 g of carbohydrates. Additionally, smoking was prohibited during the OGTT.

Patients with a fasting blood glucose level above 7.8 mmol/l underwent postprandial glycemia measurements 2 h after a meal containing 20–24 g of carbohydrates, 6–9 g of protein and 8–11 g of fat. The diagnostic criteria (WHO, 1999–2006) [6] for T2DM were fasting plasma the glucose level above 7.0 mmol/l or a glucose level above 11.1 mmol/l 2 h after an OGTT. A HbA1c level above 6.5 % was also used as a diagnostic criterion for T2DM.

### Blood sampling and biochemical assays

Venous blood was collected from all patients. Serum was separated from venous blood by centrifugation at 3000×*g* for 20 min and stored at −70 °C. At days 1 and 12 from the MI onset, serum glucose insulin and C-peptide levels. Glucose levels were measured using a standard Thermo Fisher Scientific test system (Thermo Fisher Scientific Oy, Vantaa, Finland) in a Konelab 30i biochemistry analyzer (Thermo Fisher Scientific Oy). The intra-assay coefficients of variation (CV) for insulin Diagnostic Systems Laboratories (Webster, TX, USA) and C-peptide ELISA (Waterloo, Australia) were 3.8 and 4.2 %, respectively, and the inter-assay CV were 6.9 and 7.9 %, respectively. Adipokine, leptin and adiponectin levels were measured with the BioVendor R and D Product (Brno, Czech Republic), and the intra-assay CV were 5.9 and 6.8 %, respectively.

### Statistical analysis

Statistical analysis was performed using Statistica 6.1 (InstallShield Software Corp., Chicago, IL, USA) and SPSS 17.0 for Windows (SPSS Inc., Chicago, IL, USA). The Kolmogorov–Smirnov test was used to assess the distribution of two data sets. The results were presented as the median (Me) and the 25 and 75 % quartiles (Q1;Q3). The statistical analyses were performed using the non-parametric Mann–Whitney test and the Wilcoxon test for skewed distributions. The exact Fisher’s test was used to analyze the difference in the frequencies in two independent groups with two-sided confidence intervals. Stepwise logistic regression and a receiver operating characteristic (ROC) curve with the area under the curve (AUC) measurements were used to determine the most informative visceral obesity parameters, with hazard ratios (HR) and confidence intervals (95 %). Differences were considered statistically significant at p < 0.05.

## Results

Clinical and demographic data are shown in Table 1. The mean age of the patients was 58.7 (52.2–69.9) years. The mean age of the control group was 58.42 (52.2–61.1) years. The anthropometric measurements were BMI of 24.3 (21.8–24.9) and WC of 92 cm (79–93) and a VAT/SAT ratio below 1.0, which did not exceed the range for abdominal fat at this age. The CT findings demonstrated that none of the control subjects suffered from visceral obesity (VAT area of 110.0 [104.0–128.0] cm^2^ and VAT/SAT ratio of 0.35 [0.2–0.39]).The groups were comparable in age and presence of risk factors for coronary artery disease (CAD) such as hypertension (AH), smoking, signs and symptoms of angina pectoris prior to MI, congestive heart failure and acute cerebrovascular accidents (p > 0.05). However, patients with excessive VAT had more frequent family history of T2DM and CAD, as well as a history of MI compared with patients without excessive VAT (p < 0.05). A similar prevalence of anterior Q-wave MI was found in both groups (p > 0.05). There were no significant differences between groups in terms of complication rates during hospitalization for MI and left ventricle (LV) ejection fraction (p > 0.05). Patients in both groups suffered from multivessel CAD. However, severe atherosclerotic lesions of coronary arteries were found in patients with excessive VAT (e.g., 3-vessel disease).

**Table 1:** Baseline clinical characteristics of patients

Variable	Patients with visceral obesity, n = 64	Patients without visceral obesity, n = 30	p value
Age, years	58 (54.69)	59 (50.67)	0.92
Arterial hypertension, n (%)	64 (100.0)	25 (83.3)	0.05
Current smoking, n (%)	36 (56.6)	16 (53.3)	0.46
Family history of IHD, n(%)	42 (65.6)	10 (30.0)	0.04
Family history of T2DM, n (%)	14 (21.8)	3 (10.0)	0.04
Angina prior to myocardial infarction, n (%)	34 (53.1)	20 (66.6)	0.65
Previous myocardial infarction, n (%)	12 (18.8)	5 (16.6)	0.04
History of heart failure, n(%)	6 (9.3)	3 (10.0)	0.75
History of cerebrovascular accident/transient ischemic attack, n (%)	0	1 (3.3)	1.0
Myocardial infarction			
Q-wave myocardial infarction	51 (79.6)	24 (80.0)	0.59
Non-Q-wave myocardial infarction	13 (20.4)	6 (20.0)	0.67
Localization of myocardial infarction			
Posterior	42 (65.6)	16 (53.3)	0.62
Posterior with extension to the right ventricle	7 (10.9)	4 (13.3)	0.68
Anterior	12 (18.8)	8 (26.6)	0.54
Inferio- posterio-lateral	3(4.7)	2 (6.6)	0.73
Acute heart failure (Killip classification)			
I	44 (68.7)	20 (66.6)	0.67
II	13 (20.3)	7 (23.3)	0.55
III	6 (9.3)	3 (10.0)	0.69
IV	1 (6.4)	0	1.00
Rhythm disturbance, n (%)	17 (26.6)	8 (26.6)	0.85
Early post-infarction angina, n (%)	12 (18.7)	6 (20.0)	0.74
Recurrent myocardial infarction, n (%)	3 (4.6)	1 (3.3)	0.89
Creatine phosphokinase, U/l	340.2 (213.1,7;738.4)	253 (115.7; 534.1)	0.02
Max creatine phosphokinase-MB, U/l	83 (37;178)	67 (36; 144)	0.04
Troponin T, ng/ml	1.1 (0.88:3.1)	0.71 (0.17:1.2)	0.01
Left ventricular ejection fraction, %	51 (43;58)	54 (43; 57)	0.627
Number of diseased coronary arteries			
Stenosis of a vessels	10 (15.6)	6 (20)	0.79
Stenosis of two vessels	6 (9.4)	10 (33.3)	0.03
Stenosis of three or more vessels	48 (75.0)	14 (46.6)	0.04
Treatment strategy/group of drugs			
Stenting of the infarct-related artery	64 (100)	30 (100)	0.79
Systemic thrombolytic therapy	5 (7.8)	3 (10.0)	0.76
β-Blockers	63 (98.4)	30 (100)	0.82
Angiotensin-converting enzyme	58 (90.6)	26 (86.7)	0.68
Calcium channel blocker	53 (82.8)	24 (82.8)	0.98
Diuretics	22 (34.3)	11 (36.6)	0.83
Nitrates	9 (14.0)	4 (13.3)	0.77
Aspirin	64 (100)	29 (96.6)	0.92
Heparin	64 (100)	30 (100)	0.98
Clopidogrel	57 (89.0)	27 (90.0)	0.83
Statins	64 (100.0)	30 (100.0)	0.98

p value for differences between groups (P < 0.05)

*HIS* ischemic heart disease, *T2DM* type 2 diabetes mellitus

The anthropometric measurements showed that 69 % of MI patients suffered from visceral obesity. Patients with normal weight and those with overweight were equally distributed among patients with excessive VAT (Table 2). The BMI values were lower in patients with visceral obesity compared with patients without it. Patients in Group 2, without visceral obesity, tended to have higher BMI and be overweight. Moreover, patients with grade 3 obesity were more likely to be found in this group. Abdominal obesity (WC > 94 cm) was more likely to be found in patients without visceral obesity and may be associated with increased SAT area, rather than VAT.

**Table 2:** Anthropometric indicators of obesity in patients with myocardial infarction

Variable	Patients with visceral obesity, n = 64	Patients without visceral obesity, n = 30	p value
BMI, kg/m^2^	26.7 (19.0;42.0)	29.4 (25.1;39.2)	0.02
Normal weight, n (%)	24 (37.5)	6 (20)	0.02
Overweight, n (%)	20 (31.5)	14 (46.6)	0.2
I obesity, n (%)	15 (23.4)	2 (6.7)	0.01
II obesity, n (%)	3 (4.7)	2 (6.7)	0.23
III obesity, n (%)	2 (3.1)	6 (20)	0.03
WC, cm	103.8 (84.0;124.0)	96.8 (78.0;123.0)	0.04
HC, cm	97.7 (90.0;120.0)	101.2 (84.0;140.0)	0.04
WC/HC	1.0 (0.9;1,2)	1.0 (0.7;1.1)	0.23
Area VAT, cm^2^	162.2 (130.3;196.4)	119.0 (102.1;129.2)	0.01
Area SAT, cm^2^	382.2 (236.4;435.0)	411.0 (334;470)	0.01
VAT/SAT	0.44 (0.38;0.62)	0.31 (0.18;0.55)	0.01

Data in the table are presented as median (Me) and 25 and 75 % quartiles (Q1;Q3) or n (%)

p value for differences between groups (p < 0.05)

*VAT* visceral adipose tissue, *BMI* body mass index, *SAT* subcutaneous adipose tissue, *HC* hip circumference, *WC* waist circumference

**Table 3:** Basal and postprandial carbohydrate metabolism and HOMA-IRmarkers in MI patients during hospitalization

Variable	Control group (n = 30)		Patients with visceral obesity, n = 64			Patients without visceral obesity, n = 30		
			1st day	12th day		1st day	12th day	
	Basal level	Postprandial level		Basal level	Postprandial level		Basal level	Postprandial level
	1	2	3	4	5	6	7	8
Glucose, mmol/l	5.05 (4.9;5.4)	5.35 (5.1;5.7)	7.8 (4.3;9.9) p_1–3_ = 0.01	7.1 (5.7;10.8) p_1–4_ = 0.02	7.85 (5.9;9.7) p_2–5_ = 0.05	7.5 (5.4;8.7) p_1–6_ = 0,01	5.7 (5.2;7.6) p_4–7_ = 0.02 p_6–7_ = 0.02	6.0 (4.9;7.2) p_2–8_ = 0.04 p_5–8_ = 0.04
Insulin, mU/ml	9.62 (7.6;12.2)	16.5 (14.2;18.9)	14.7 (9.6;23.2) p_1–3_ = 0.003	11.6 (5.7;19.5) p_3–4_ = 0.02 p_1–4_ = 0.02	46.88 (10.55;56.63) p_2–5_ = 0.02	10.3 (7.8;13.0) p_1–6_ = 0.04 p_3–6_ = 0.02	8.9 (4.5;14.5) p_4–7_ = 0.02 p_6–7_ = 0.04	23.09 (4.36;47.61) p_2–8_ = 0.04 p_5–8_ = 0.01
C-peptide, ng/ml	1.7 (1.41;2.3)	3.2 (2.9;3.3)	2.3 (1.1;2.7) p_1–3_ = 0.02	1.9 (0.9;2.5) p_3–4_ = 0.02 p_1–4_ = 0.03	5.1 (1.5;5.7) p_2–5_ = 0.00	1.8 (0.4;1.9) p_3–6_ = 0.01	1.6 (0.7;1.8) p_4–7_ = 0.04	3.5 (2.8;6.1) p_2–8_ = 0.03 p_5–8_ = 0.03

Data in the table are presented as median (Me) and 25 % and 75 % quartiles (Q1;Q3). *P* value for differences between groups (p < 0.05)

*MI* myocardial infarction, *HOMA-IR* homeostasis model assessment method for the evaluation of IR

Taking into consideration the close pathogenetic relationship between obesity and IR, the IR markers (insulin levels, C-peptide levels and HOMA-IR) and basal and postprandial glucose levels were measured on days 1 and 12 from MI onset. Levels of carbohydrate metabolism markers are shown in Table 3. In both groups of patients as compared with the control group, elevated glucose levels were found on day 1 of the MI treatment (Table 3). Further, patients with visceral obesity had a constantly elevated glucose level during the entire follow-up period. However, in patients without visceral obesity, glucose levels decreased on day 12 (p > 0.05) to similar values compared with the control subjects.

High glucose levels in patients with visceral obesity were accompanied by an increase in HOMA-IR values, insulin and C-peptide levels compared with patients without visceral obesity. On day 1 of the MI treatment, basal insulin levels, C-peptide levels and HOMA-IR values increased 1.42-, 1.28- and 1.6-fold, respectively, in comparison with patients without visceral obesity (Tables 3, 4). There was a decrease in insulin and C-peptide levels in both groups on day 12. Patients with visceral obesity presented 1.34 times higher insulin levels compared with patients without visceral fat. HOMA-IR values did not change significantly in patients with excessive VAT on day 12, while in subjects without visceral obesity, these values decreased and reached similar values compared with the control subjects.

**Table 4:** Adipokine status indicators in patients with myocardial infarction with or without visceral obesity during hospitalization

Variable	Control (n = 30)	Patients with visceral obesity, n = 64		Patients without visceral obesity, n = 30	
		1st day	12th day	1st day	12th day
	1	2	3	4	5
Leptin, ng/ml	5.1 (4.6;5.3)	29.3 (15.0;41.4) p_1–2_ = 0.01	20.07 (14.4;30.1) p_1–3_ = 0.01 p_2–3_ = 0.001	14.6 (9.1;22.0) p_1–4_ = 0.002 p_2–4_ = 0.001	9.7 (5.8;10.7) p_1–5_ = 0.005 p_3–5_ = 0.004 p_4–5_ = 0.003
Adiponectin, mg/ml	13.3(9.4;14.5)	7.7 (5.4;10.6) p_1–2_ = 0.01	8.3 (5,3;11.2) p_1–3_ = 0.02 p_2–3_ = 0.04	9.7 (8.5;13.0) p_1–4_ = 0.02	12.3 (8.7;16.3) p_1–5_ = 0.04 p_3–5_ = 0.02 p_4–5_ = 0.04

Data in the table are presented as median (Me) and 25 and 75 % quartiles (Q1;Q3)

p value for differences between groups (p < 0.05)

During the MI treatment, an increase in basal and postprandial glucose levels as well as in insulin and C-peptide levels was found. However, more pronounced disorders were found in patients with visceral Postprandial glucose, insulin and C-peptide levels were 1.31-, 2.0- and 1.45-fold higher, respectively, in Group 1 than in Group 2. Postprandial glucose and insulin were 1.6- and 2.8-fold higher than in the control group (p < 0.05), respectively. Notably, there were no significant differences in C-peptide levels between both groups compared with the control subjects.

Adipokines have a primary role in regulating carbohydrate metabolism. Although all MI patients presented an imbalance in the adipokine status indicators, these changes were far more pronounced in patients with visceral obesity (Table 4). On day 1 from the onset of MI, patients in Group 1 showed a 2.0-fold increase in leptin levels compared with patients without visceral obesity and a 5.7-fold increase compared with the control group. On day 12, patients with excessive VAT reported a 1.4-fold decrease in leptin levels.

Importantly, adiponectin levels decreased by 1.7-fold in patients with visceral obesity on day 1 from the onset of MI compared with control subjects, and decreased by 1.3-fold compared with patients without visceral obesity. On day 12 of the MI treatment, adiponectin remained at low levels in Group 1, whereas in Group 2, the adiponectin levels increased nearly to the baseline values.

Carbohydrate disorders and adipokine imbalance in patients with visceral obesity were associated with the onset of T2DM 1 year after MI. Diabetes was diagnosed in 11 patients in Group 1. Moreover, a further 11 patients in this group presented impaired glucose tolerance (IGT) and six patients had abnormal fasting glucose levels. Interestingly, there were no such findings in patients without visceral obesity.

Univariate logistic regression and ROC-analysis were performed to determine the prognostic value of anthropometric measurements, biochemical results and biomarkers for the risk assessment of developing diabetes 1 year after MI (Table 5). The results showed that the VAT area had the highest predictive value among the anthropometric measurements. Patients with a VAT area equal to or above 130 cm^2^ were at a 3.6-fold higher risk of developing T2DM (AUC 0.91, p = 0.00). The BMI did not yield a high diagnostic accuracy. The HOMA-IR and the leptin levels measured on days 1 and 12 showed the highest diagnostic accuracy among the markers of carbohydrate metabolism and adipokine status assessed.

**Table 5:** Odds ratios for diabetes based on anthropometric and biochemical parameters 1 year after myocardial infarction

Variable	OR	95 % CI	AUC	p value
BMI	1.3	1.0–1.1	0.55	0.07
WC	1.7	1.4–1.9	0.76	0.01
HC	1.6	1.4–1.8	0.72	0.01
WC/HC	1.9	1.8–2.3	0.84	0.01
VAT area, cm^2^	3.6	2.2–4.2	0.91	0.00
SAT area, cm^2^	1.1	0.9–1.2	0.55	0.09
VAT/SAT	1.6	1.4–1.8	0.72	0.01
Basal level glucose, 1st day, mmol/l	1.2	1.1–1.4	0.72	0.01
Basal level glucose, 12th day, mmol/l	1.3	1.1–1.5	0.74	0.03
Postprandial level glucose, 12th day, mmol/l	1.9	1.6–2.1	0.82	0.00
Basal level insulin, 1st day, mU/ml	1.2	1.1–1.4	0.72	0.02
Basal level insulin, 12th day, mU/ml	1.3	1.1–1.5	0.77	0.04
Postprandial level insulin, 12th day, mU/ml	1.6	1.2–1.8	0.79	0.01
Basal level C-peptide, 1st day, ng/ml	1.2	1.2–1.6	0.72	0.03
Basal level C-peptide, 12th day, ng/ml	1.5	1.1–1.5	0.76	0.03
Postprandial level C-peptide, 12th day, ng/ml	1.7	1.5–1.9	0.80	0.01
HOMA-IR, 1st day	1.8	2.9–4.1	0.75	0.01
Index HOMA-IR, 12th day	3.5	2.8–3.9	0.93	0.01
Adiponectin, 1st day, mg/ml	0.7	0.6–0.8	0.74	0.01
Adiponectin, 12th day, mg/ml	0.6	0.5–0.8	0.76	0.02
Leptin, 1st day, ng/ml	3.7	2.9–4.0	0.96	0.01
Leptin, 12th day, mg/ml	3.1	2.8–3.6	0.90	0.01

p value for differences between groups (P < 0.05)

*BMI* body mass index, *VAT* visceral adipose tissue, *SAT* subcutaneous adipose tissue, *HC* hip circumference, *WC* waist circumference, *HOMA-IR* homeostasis model assessment method for the evaluation of IR, *OR* odds ratio, *CI* confidence interval, *AUC* area under the curve

Thus, patients with visceral obesity demonstrated unfavorable prognosis following MI, i.e., the manifestation of T2DM in the long-term post-MI period. The biochemical basis of the pathological process is presented with basal and postprandial hyperglycemia, hyperinsulinemia and IR, as well as adipokine imbalance in acute MI. Moreover, it was more pronounced in patients with visceral obesity.

## Discussion

Obesity is a commonly recognized risk factor of T2DM [1]. However, there are no informative indicators to assess the risk of developing T2DM in MI patients who are overweight in clinical practice [9].

BMI and WC are routinely used to assess overweight. Further, it is well known that these parameters are correlated with the risk of developing CAD and diabetes. WC is strongly associated with the risk of developing new-onset diabetes, as it reflects fat accumulation in the abdominal area, which is considered to be particularly unfavorable for developing cardiometabolic complications. However, in the present study, BMI had low value in terms of predicting the development of T2DM, with high BMI representing a 1.3-fold increased risk of developing diabetes. Interestingly, WC and waist-to-hip ratio were far more valuable indicators. Abnormalities in these parameters represented a 1.7- and 1.9-fold, respectively, increased risk of developing manifestations of diabetes. Nevertheless, we found that the most valuable indicator of obesity associated with the risk of developing T2DM was the VAT area in the abdominal compartment, meaning that the presence of excessive visceral fat represents a 3.6-fold increased risk of developing T2DM. VAT area also had higher sensitivity compared with the other markers of abdominal obesity.

Low informative values of BMI and WC may be explained by the fact that these parameters do not account for the ratio of muscle, bone, fat mass and the distribution of adipose tissue. As VAT indicates the visceral fat area in the abdominal compartment, it therefore, represents the highest risk factor for developing CVD and T2DM. In this study, more than half of the MI patients suffered from visceral obesity according to the CT scan findings. The BMI in patients with visceral obesity was significantly lower than in those patients without visceral obesity. Similar results were presented in 2008 by Oreopoulos et al. [10], who showed that BMI in patients with chronic heart failure correctly reflected the amount of adipose tissue in 41 % of examined patients. In their cohort of middle-aged (62–66 years) normal-weight, overweight and obese patients, BMI was a better indicator of lean body mass than of adiposity [10].

Excessive accumulation of visceral fat is a well-known indicator of obesity-related cardiometabolic changes [5]. In our study, visceral obesity was associated with multivessel CAD and severe damage of cardiomyocytes during MI, as evidenced by elevated CK-MB levels in patients with visceral obesity. Excessive VAT is associated with higher levels of metabolic activity of adipocytes compared with SAT. The synthesis of fat and biologically active hormone-like substances is more intense in VAT adipocytes. In contrast to SAT adipocytes, visceral fat cells are less sensitive to the effects of insulin and are more susceptible to catecholamines, which stimulate lipolysis and are especially important during catecholamine stress, common in MI [11]. Intensive lipolysis in visceral adipocytes leads to excessive release of free fatty acids in the hepatic portal circulation, entering the liver and decreasing the sensitivity of insulin receptors on hepatocytes. These events lead to impaired hepatic insulin clearance and the development of systemic hyperinsulinemia.

Hyperinsulinemia enhances IR by violating autoregulation in muscle insulin receptors. Our study results showed a 1.5-fold increase in HOMA-IR, other IR markers (insulin and C-peptide) and glucose in patients with visceral obesity during the hospitalization period compared with the healthy volunteers and the MI patients without excessive VAT. The most informative parameters for assessing the risk of developing T2DM were HOMA-IR, postprandial hyperglycemia and hyperinsulinemia.

Adipokines have a pivotal role in the development and progression of IR and its various manifestations during MI. They have a variety of local, peripheral and central effects, affecting food intake, metabolic processes, and oxidative stress in the cardiovascular system [12]. The present results indicate that there is an adipokine imbalance in patients suffering from visceral obesity. Such patients presented increased leptin levels and decreased serum adiponectin levels during MI hospitalization compared with the groups of control subjects and patients without visceral obesity (p < 0.05). High leptin levels were associated with a 3.0-fold increased risk of developing T2DM in the post-MI period. Adipokine imbalance was found in patients without visceral obesity on day 1 of the MI treatment, which seemed to subsequently stabilize throughout the hospitalization. Previously, patients with MI were known to have higher serum levels of leptin regardless of body weight when compared with healthy subjects [13, 14]. These results may be associated with the activation of alternative sources of leptin in MI. Cardiomyocytes are known to be able to express adipokines, which apparently may be involved in regulating metabolism during MI. According to the experimental data, both endothelin-1 and angiotensin II increased leptin levels in cardiomyocyte cultures [15]. We found a high concentration of leptin in acute MI, which suggests ischemia-induced leptin production by adipocytes and cardiomyocytes. Further, a highly active VAT contributes to the total amount of leptin, which seems to limit the area of necrosis and contribute to myocardial regeneration in the acute and early recovery phases of MI.

However, elevated blood levels of leptin are known to be closely associated with the development of IR, which is not only a sign of T2DM, but also a metabolic risk factor of atherosclerosis and MI [12]. Wallander et al. [16] suggested that high serum leptin levels are a predictor of both repeated cardiovascular events and the development of impaired carbohydrate tolerance in patients with MI. One of the pathophysiological mechanisms of this phenomenon is the ability of leptin to modulate the cell signaling pathways involved in the insulin, carbohydrate, lipid and energy metabolisms, resulting in increased myocardial injury during ischemia [12].

Adiponectin levels decrease during MI compared with leptin levels. The concentration of adiponectin within the normal range is usually determined by the size and the number of visceral adipocytes, which produce more adiponectin than subcutaneous adipocytes. Paradoxically, obesity is characterized by decreased serum adiponectin levels, which is apparently provoked by decreased adiponectin secretion [17]. Adiponectin production is reduced with obesity, which may be associated with the inhibition of adiponectin gene transcription by inflammatory angiogenic factors produced by hypertrophic adipocytes. According to our results, adiponectin levels in patients without visceral obesity tended to be within the normal range on day 12 of MI treatment, whereas in patients with visceral obesity, adiponectin levels remained significantly reduced. According to our results, the deficiency of adiponectin during the hospitalization period of MI increases the risk of diabetes by 30 %.

## Conclusion

Visceral obesity in patients with MI is associated with more severe IR, leptin and adiponectin imbalance, and development of IGT and T2DM. Diagnostic criteria of obesity have different predictive values regarding the risk of developing T2DM. The VAT area was the indicator with the highest and BMI the indicator with the lowest diagnostic accuracy. Our results indicate there is a need for a differentiated approach in the diagnosis of obesity based on adipose tissue distribution. The CT assessment of the adipose tissue distribution seem to have higher predictive value than BMI. Further information regarding predictors of a higher risk of cardiovascular events and T2DM will assist in initiating proper therapeutic and preventive interventions in patients with visceral obesity.

## Abbreviations

WC: waist circumference
BMI: body mass index
VAT: visceral adipose tissue
IR: insulin resistance
CVD: cardiovascular disease
T2DM: type 2 diabetes mellitus
MI: myocardial infarction
CT: computed tomography
HC: hip circumference
SAT: subcutaneous adipose tissue
OGTT: oral glucose tolerance test
CAD: coronary artery disease
IGT: impaired glucose tolerance
